# A Pathway Model to Understand the Evolution of Spike Protein Binding to ACE2 in SARS-CoV-2 Variants

**DOI:** 10.3390/biom12111607

**Published:** 2022-10-31

**Authors:** Ludovico Pipitò, Christopher A. Reynolds, Juan Carlos Mobarec, Owen Vickery, Giuseppe Deganutti

**Affiliations:** 1 Centre for Sport, Exercise and Life Sciences, Faculty of Health and Life Sciences, Coventry University, Coventry CV1 5FB, UK; 2 School of Life Sciences, University of Essex, Wivenhoe Park, Colchester CO4 3SQ, UK; 3 Mechanistic and Structural Biology, Discovery Sciences, R&D, AstraZeneca, Cambridge, UK

**Keywords:** SARS-CoV-2, spike protein ACE-2, molecular dynamics, supervised molecular dynamics, binding pathway

## Abstract

After the SARS-CoV-2 Wuhan variant that gave rise to the pandemic, other variants named Delta, Omicron, and Omicron-2 sequentially became prevalent, with mutations spread around the viral genome, including on the spike (S) protein; in order to understand the resultant in gains in infectivity, we interrogated in silico both the equilibrium binding and the binding pathway of the virus’ receptor-binding domain (RBD) to the angiotensin-converting enzyme 2 (ACE2) receptor. We interrogated the molecular recognition between the RBD of different variants and ACE2 through supervised molecular dynamics (SuMD) and classic molecular dynamics (MD) simulations to address the effect of mutations on the possible S protein binding pathways. Our results indicate that compensation between binding pathway efficiency and stability of the complex exists for the Omicron BA.1 receptor binding domain, while Omicron BA.2′s mutations putatively improved the dynamic recognition of the ACE2 receptor, suggesting an evolutionary advantage over the previous strains.

## 1. Introduction

The *equilibrium* binding of novel strains of the severe acute respiratory syndrome coronavirus 2 (SARS-CoV-2) to the ACE2 receptor has been extensively studied [1] as part of the quest to understand the enhanced infectivity of variants of concern (VOC); here we have used the supervised molecular dynamics (SuMD) method [2] to focus on differences in the binding *pathway* of Delta and Omicron variants. The Omicron strain of SARS-CoV-2, B.1.1.529 that originated in South Africa (https://www.gisaid.org/phylodynamics/west-africa/ (accessed on 11 June 2022)) was identified by the World Health Organization (WHO) on the 24 November 2021 and was declared a variant of concern (VOC) two days later [3]. Various Omicron cases were reported in travellers from South Africa, followed by reports from people around the world [4,5], raising concerns amongst the scientific community and governments alike. Omicron cases in South Africa, America, and India reached their peak in January 2022 right after the Delta variant started to be under control [6], with a similar scenario in Europe where Delta and Omicron continued to compete, while further new Omicron variants, e.g., BA.2, BA.2.75, BA.3, BA.4, and BA.5 were observed [6,7].

**Figure 1:**
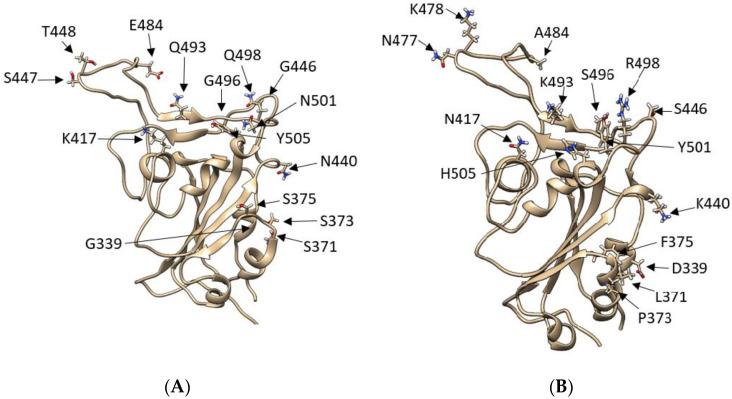
Comparison between SARS-CoV-2 RBD wild type and SARS-CoV-2 RBD Omicron: (**A**) SARS-2 WT RBD model showing residues T333-P527, with the original WT amino acids represented as liquorice. (**B**) SARS-2 WT RBD model showing residues T333-P527, with the Omicron mutations highlighted and represented as liquorice. With a total of 50 mutations, 15 of which are on the RBD, the Omicron variant possesses a different configuration of polar residues in the region between N477-H505 and a notable K417N mutation.

The presence of more than 50 mutations (Figure 1, Table S1), including deletions, raised concerns and speculations about Omicron’s ability to evade the innate immune response. Mutations K417N, G446S, S477N, T478K, E484A, Q493R, G496S, Q498R, N501Y, and Y505H are part of the immunodominant antigenic site I [8]. Despite concerns about Omicron and its evasion mechanism, experimental data indicated that Omicron’s mutations heavily impact the viral replication and pathogenicity through inefficiency in exploiting the cellular serine protease TMPRSS2 [9] compared to Alpha, Beta, and Delta variants. In Shuai’s experiments, mice infected with Omicron showed a drastic reduction in viral replication and a strongly reduced pro-inflammatory response as indicated by the modest gene expression of the interferon-gamma induced protein 10 (IP-10) and a reduced interferon-gamma production (IFNγ). The mutations on the S1/S2 domain and the N-terminal Domain (NTD) suggested the intriguing hypothesis that Omicron could compromise the cell’s ability to degrade its viral components while also reducing the efficacy of the majority of the vaccines [10] due to 15 mutations present on the receptor-binding domain [11] (RBD, Table S1). Some of these mutations are conserved between Beta and Delta strains [12]. According to mutational scanning experiments, almost all the mutations involving the RBD did not increase ACE2 binding affinity when present individually [13], while only the N501Y or L452R mutation enhances the binding to ACE2, by 6-fold or more [14,15,16,17]; this is due to increased shape complementarity with the Y41^ACE2^ and K353^ACE2^ side chains in the case of N501Y. Mutations Q493K (or Q493R) and Q498R introduced new ionic interactions with E35^ACE2^ and E38^ACE2^ but displayed slightly reduced avidity when tested individually in the yeast-displayed SARS-CoV-2 RBD [13]. The K417N mutation, on the other hand, worsens ACE2 recognition by about 3-fold [14,18] through the loss of a salt bridge with D30^ACE2^, although the effect on the binding when combined with other mutations is reportedly smaller [19]. Notably, all these Omicron mutations seem to compensate each other when it comes to the binding affinity for ACE2, as the evidence suggests that the RBD of Omicron BA.1 (RBD°) has a binding energy similar to that of the other strains [19,20,21]. However, a recent surface plasmon resonance study showed that the Omicron RBD has a higher affinity for ACE2 than WT and Delta [22].

In the present work, we first interrogated the RBDs from Wuhan (wild type, WT), Delta, and Omicron variants in complex with the ACE2 ectodomain using molecular dynamics (MD) simulations and proposed a unique network of hydrogen bonds characterising Omicron. We then studied the out-of-equilibrium process of Delta RBD (RBD^Δ^) or RBD° binding to ACE2, employing supervised MD [2,23] (SuMD) simulations. We propose that the same mutations stabilising the equilibrium Omicron complex with ACE2 hamper the binding pathway and the kinetics of binding, accounting for the overall compensation on the measured affinity for the receptor.

## 2. Methods

### 2.1. Structure Preparation and Force Field Settings

All systems were prepared using the CHARMM36 [24,25]/CGenFF 3.0.1 [26,27] force field combination. The model of the RBD° of the S protein RBD model was modelled through alphafold2 [28] and comprised residues T333-P527. RBD^Δ^ was retrieved from PDB ID 7V8B. ACE2 residues S19 to A614 were obtained from PDB ID 6M17. RBD^BA.2^ was modelled by introducing the following mutations L371F, T376A, D405N, R408S, S446G, and S496G in the RBD° model.

The protonation state of residue side chains was calculated on the dissociated ACE2-RBD complex before SuMD binding simulations (see Methods section: Supervised molecular dynamics) by Propka [29] at a simulated pH of 7.45 and added by pdb2pqr [30]. This step was performed primarily to identify any closely coupled charged residue clusters that required anomalous pKas. Only exposed residues D206^ACE2^, E375^ACE2^, and E489^ACE2^ were predicted in a non-canonical protonated state. We visually inspected these residues and assigned them to the unprotonated form on the basis of the surrounding environment and solvent-accessible surface area (Figure S1). As a control, we used Schrödinger’s Protein Preparation Wizard, which predicted E375^ACE2^ and E489^ACE2^ in the canonical unprotonated state. Disulphide bonds were identified by HTMD [31], visually inspected, and patched manually through VMD [32]. The initial geometry and internal energy were optimised using ACEMD [33].

### 2.2. System Preparation for Classic Molecular Dynamics

The RBD^WT^:ACE2 complex from PDB 6M17, as per our previous work [23], was used as a reference for the preparation of both RBD^Δ^:ACE2 and RBD°: ACE2 complexes. RBD^Δ^:ACE2 was obtained by superimposing RBD^Δ^ from PDB 7V8B onto RBD^WT^:ACE2, while the RBD°: ACE2 complex was obtained by superimposing an RBD° model obtained by AlphaFold2 [28] on the RBD^WT^:ACE2 complex. Glycan residues and the Zn^2+^ cation were removed, topology files were prepared using VMD’s Psfgen plugin (https://www.ks.uiuc.edu/Research/vmd/plugins/psfgen/ (accessed on 9 January 2022)), and the resulting structures were visually inspected after their creation. The systems were simulated for a total time of 500 ns in triplicate with TIP3P water molecules [34] were added to the simulation box using the Solvate plugin 1.5 (http://www.ks.uiuc.edu/Research/vmd/plugins/solvate/ (accessed on 9 January 2022)) to give a 15 Å padding in every direction. The charge neutrality was achieved by adding Na^+^/Cl^−^ to the concentration of 0.150 M using the Autoionize plugin 1.3 (http://www.ks.uiuc (accessed on 9 January 2022)). ACEMD was used for both the equilibration and the productive MD trajectories (Table S2). The energy of the systems was reduced through 1000 conjugate-gradient minimisation steps to eliminate possible clashes and optimise atomic distances. Equilibration was reached in isothermal-isobaric conditions (NPT) using the Berendsen barostat [35] (target pressure 1 atm) and the Langevin thermostat [36] (target temperature 310 K) during a 4 ns long MD simulation (integration time step 2 fs). During the equilibration, a positional restraint of 1 kcal mol^−1^ Å^2^ was applied on the alpha carbons of both ACE2 and the RBD for the first 3 ns, and on protein side chains for the first 2 ns. Productive trajectories were produced in triplicate with an integration time step of 4 fs, using hydrogen mass repartition [37] in the canonical ensemble (NVT), with no positional restraints. The cut-off distance for electrostatic interactions was set at 9 Å, with a switching function applied beyond 7.5 Å. Long-range Coulomb interactions were handled using the particle mesh Ewald summation method (PME) [38] with default ACEMD settings. Atomic velocity was reassigned in each replicate to increase the sampling and explore possible alternate conformations.

### 2.3. Supervised Molecular Dynamics (SuMD)

SuMD is an adaptive sampling method [39] for speeding up the simulation of binding [2,40] and unbinding processes [41]. During SuMD, sampling is gained without the input of any energetic bias, by applying a “tabu–like” algorithm to monitor the distance between centres of mass (or geometrical centres) chosen on ligand and receptor. Consecutive unbiased short MD simulations are performed, and, after each simulation, the distances (collected at regular time intervals) are fitted to a linear function. If the slope of the linear fitting function is negative, then the next short MD will start from the last coordinates and velocities, otherwise, the simulation will be restarted by randomly assigning the atomic velocities according to the Boltzmann distribution [42].

An initial distance between RBD^WT^ and ACE2 surfaces was set at 25 Å, allowing conformational exploration during the binding pathway as per our previous work [23]. The initial position of RBD^Δ^ or RBD° was obtained by superimposing them on the RBD^WT^ through Chimera’s align feature, producing identical starting conditions. Ultimately, the topology files were built through VMD’s Psfgen and visually inspected. TIP3P water molecules were added to the simulation box to achieve a 15 Å water padding, using the minimum and maximum coordinates of the structures as a reference. The charge neutrality of the system was achieved by adding Na^+^/Cl^−^ to the concentration of 0.150 M using the Autoionize plugin 1.3. Eight independent replicas of SuMD were produced for the Delta and Omicron RBD. The simulations were produced by supervising the distance between the RBD Cα atoms of residue K31^ACE2^ and Q493^Δ^ or K493° on the RBD “up” binding motif (RBD). A series of 600 ps long time windows were simulated until the supervised distance reached a value lower than 10 Å without further improvements in the distance. Frames were saved every 200 ps and used to interpolate the linear function of the distance during the simulated 600 ps. For each replica, a 200 ns long MD trajectory was produced starting from the last coordinate produced by SuMD to relax the system without any supervision.

### 2.4. MD Trajectories Analysis

Out of eight SuMD binding replicas collected for RBD^Δ^, RBD°, and RBD^BA.2^ (Table S2), the best three (RBD° and RBD^BA.2^) or four (RBD^Δ^) replicas in terms of final root mean square deviation (RMSD) to the experimental complex geometry were analysed. 

RMSD analyses were computed using VMD and MDTraj [43]. Ligand-protein contacts, including hydrogen bonds, were detected using the GetContacts scripts tool (https://getcontacts.github.io (accessed on 9 January 2022)), with a threshold distance and angle of 3.5 Å and 120°, respectively. Contacts and HB were expressed as occupancy (% of total MD frames). The molecular mechanic energy combined with the generalised Born surface area (MM-GBSA) was computed with the MMPBSA.py [44] script (AmberTools20 suite at http://ambermd.org/, converting the CHARMM psf topology files to Amber prmtop format through ParmEd (http://parmed.github.io/ParmEd/html/index.html (accessed on 9 January 2022)).

The PBSA analysis (Figure S2) was performed on equilibrated RBD^WT^ and RBD° structures using the APBS [45] web server at (https://server.poissonboltzmann.org/ (accessed on 16 August 2022)). Principal component analysis (PCA) was computed on the Cα atoms of ACE2 and the RBDs using the Prody package [46] with default settings.

## 3. Results and Discussion

### 3.1. Omicron Mutations Strengthen the RBD Interaction with ACE2 Compared to Wild-Type and Delta 

**Figure 2:**
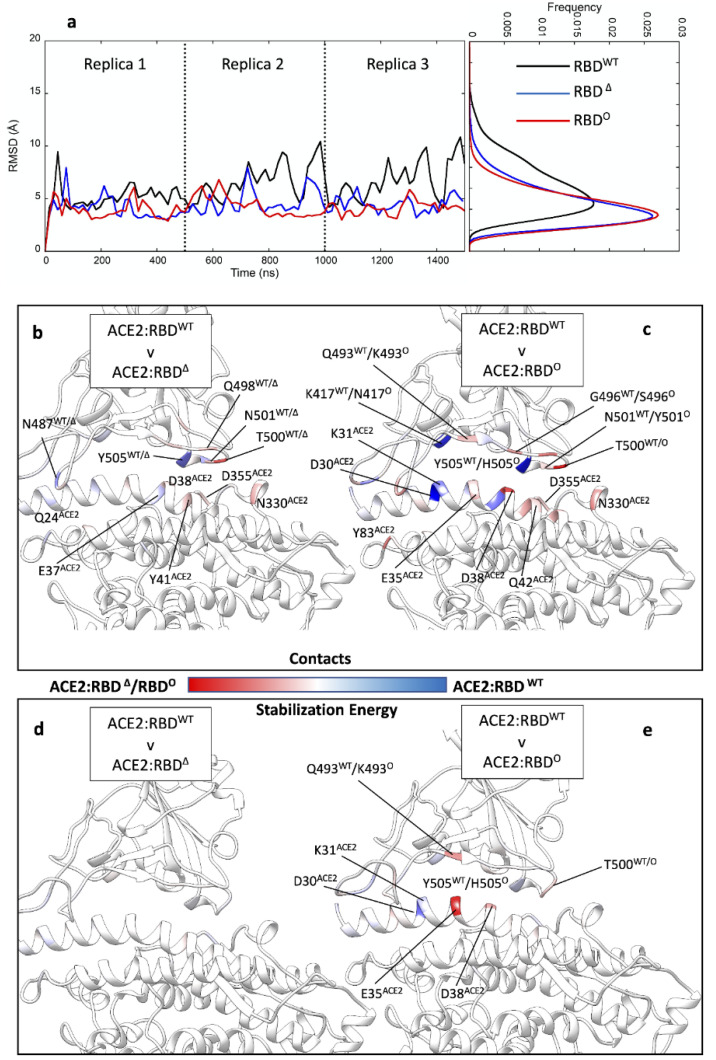
MD of ACE2 in complex with RBD^WT^, RBD^Δ^, and RBD°. (**a**) RMSD of RBD^WT^, RBD^Δ^, and RBD° over the time course of three 500 ns-long replicas (left panel, the curves were smoothed to interpolate the RMSD values) and the relative frequency distribution. (**b**) Comparison between the intermolecular contacts formed in ACE2:RBD^WT^ and ACE2:RBD^Δ^ complexes; red residues interacted more in ACE2:RBD^Δ^, while blue residues were more engaged in ACE2:RBD^WT^. (**c**) Comparison between the intermolecular contacts formed in ACE2:RBD^WT^ and ACE2:RBD° complexes; red residues interacted more in ACE2:RBD°, while blue residues were more engaged in ACE2:RBD^Δ^. (**d**) Comparison between the per residue interaction energy in ACE2:RBD^WT^ and ACE2:RBD^Δ^ complexes; red residues stabilised ACE2:RBD^Δ^, while blue residues stabilised more ACE2:RBD^Δ^. (**e**) Comparison between the per residue interaction energy in ACE2:RBD^WT^ and ACE2:RBD° complexes; red residues stabilised ACE2:RBD^Δ^, while blue residues stabilised more ACE2:RBD°.

During the preparation of this manuscript, the cryo-EM structure of the Omicron S protein in a complex with ACE2 was released [47]. We assessed the quality of our model by measuring the RMSD of RBD° to this experimental structure during MD simulations of the complex with ACE2, which resulted in a root mean square deviation (RMSD) of 3.6 ± 0.2 Å, in line with all the ACE2:RBD complexes we simulated (Figure 2a). This is close to the nominal resolution (2.45 Å to 3.40 Å) of the available RBD° structures (PDB codes: 7T9L, 7T9K, and 7WBL), indicating the validity of the AlphaFold2 model. 

We evaluated the binding properties of RBD^WT^, RBD^Δ^, or RBD° in complex with ACE2 through MD simulations, each one in triplicate (Table S2, Figure 2). RBD^Δ^ and RBD° showed similar thermal fluctuations, while the RBD^WT^ resulted in more flexibility when bound to ACE2 (Figure 2a). This is in line with previous work suggesting higher dynamicity of the RBD^WT^ compared to the Omicron structure [48]. Overall, all three different RBDs were involved in very similar rocking movements on the surface of ACE2 (Video S1). Mutations characterising RBD^Δ^ did not modify the interaction pattern with ACE2 observed for RBD^WT^ (Figure 2b), although residues common to both strands formed a different number of contacts in the two complexes. Thus, Q498^Δ^, T500^Δ^, and N501^Δ^ engaged ACE2 more than the RBD^WT^, while the WT interacted more through N487^WT^ and Y505^WT^. This slightly asymmetric interaction pattern in the contact analysis does not mirror the MM-GBSA energy analysis (Figure 2d), which shows essentially no differences in the per residue stabilisation energy and thus a high similarity between RBD^WT^ and RBD^Δ^. This high similarity is also reflected in the computed binding energies (Table S3) of −23.4 ± 4.0 kcal mol^−1^ for RBD^WT^:ACE2 and −23.0 ± 4.6 kcal mol^−1^ for RBD^Δ^:ACE2, respectively. 

Simulations of RBD° suggested a substantial change in the interaction pattern with ACE2 compared to RBD^WT^ (Figure 2c). RBD° residues R498°, K493°, S496°, Y501°, and T500° formed stable contacts with the receptor. N501Y is particularly important for the RBD affinity towards ACE2 [15,16]. RBD^WT^ was more engaged at the level of Y505^WT^ (H505° in RBD°) and K417^WT^ (N417° in RBD°). All the other mutated residues characterising RBD° (Table S1) did not participate in hydrogen bonds with the ACE2 receptor. From an energetic perspective, K493° was able to form stabilising salt bridges with ACE2 residues E35^ACE2^ and D38^ACE2^ (Table S4), compensating for unfavourable interactions with D30^ACE2^ and K31^ACE2^. The computed binding energy of the RBD°: ACE2 complex was −28.5 ± 3.4 kcal mol^−1^ (Table S3), about 5 kcal mol^−1^ more stable than both RBD^WT^:ACE2 and RBD^Δ^:ACE2, probably due to the specific electrostatic interactions involving K493°. Both RBD^WT^ and RBD^Δ^ bear a glutamine residue in position 493 (Q493^WT/Δ^) that formed a hydrogen bond with E35^ACE2^. Our MM-GBSA binding energy results are consistent with Rajender et al. [49] and Lupala et al. [50] but in disagreement with findings from other computational groups [51,52] that suggested RBD^Δ^ is a stronger ACE2 binder than RBD^WT^ or RBD°. Overall, in our simulations the total hydrogen bond occupancy for RBD^Δ^ and RBD° was 461.9 and 515.0, respectively, indicating a possible role of the electrostatic interactions in driving RBD° binding to ACE2. This is supported by single-point Poisson–Boltzmann surface area energy computations on RBD^WT^ and RBD° (Figure S2) and the electrostatic contribution to the MM-GBSA binding energy, which was −897.7 ± 89.7 kcal mol^−1^ for RBD^WT^, −1035.9 ± 96.7 kcal mol^−1^ for RBD^Δ^, and −1386.1 ± 88.3 kcal mol^−1^ for RBD°.

However, discrepancies with previous work could arise from the different lengths of the simulations or the divergent number of replicas. We averaged the results over three independent replicas, for a total simulation time of 300 ns, while other groups used a single replica. Other aspects such as the force field and the GBSA parameters employed should have a limited influence on the output [53].

### 3.2. Mutations Affect the RBD Binding Path to ACE2

**Figure 3:**
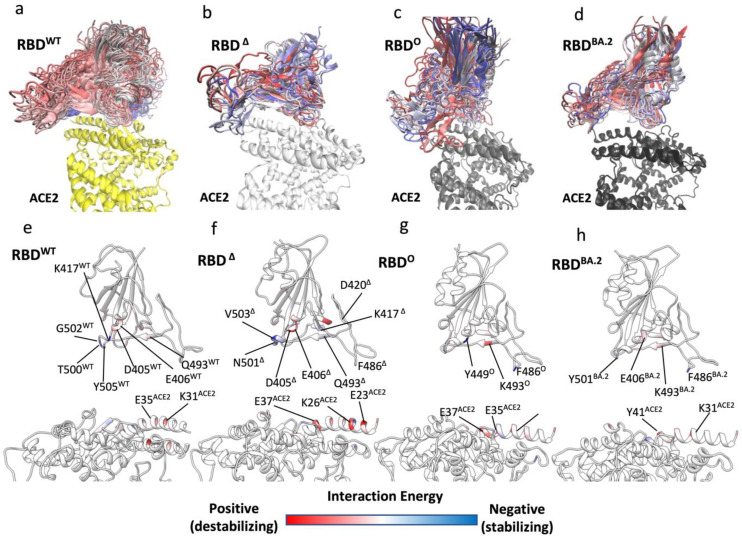
SuMD binding of RBD^Δ^, RBD°, and RBD^BA.2^ to ACE2: (**a**–**d**) Snapshots of ACE2:RBD^WT^, ACE2:RBD^Δ^, RBD°, and RBD^BA.2^ unstable complexes (MM-GBSA energy > 5 kcal mol^−1^) from SuMD replicas. RBD^WT^, RBD^Δ^, RBD°, and RBD^BA.2^ are coloured from blue to red to distinguish different frames; ACE2 in complex with RBD^WT^ is represented by a yellow ribbon. (**e**–**h**) Per residue energy decomposition of RBD^WT^, RBD^Δ^, RBD°, and RBD^BA.2^ in the unstable states from SuMD binding simulations to ACE2; only frames with binding energy > 5 kcal mol^−1^ were analysed.

Surface plasmon resonance (SPR) binding assays quantified the RBD° binding affinity for ACE2 being either 2.4-fold higher [20] than RBD^WT^ or unchanged [47], with relative differences between RBD^Δ^ and RBD° in the range of 1- to 3-fold [21,47] in favour of the latter. Surprisingly, such similar binding affinities indicate similar binding properties between RBD variants, despite the high number of mutated residues on RBD°, in contrast with the higher infectivity displayed by the Omicron variant. Therefore, we further investigated the RBD binding properties by means of SuMD, an energetically unbiased out-of-equilibrium MD technique. The goal was to study the first step of the molecular recognition between ACE2 and RBD^WT^, RBD^Δ^ (Video S2), or RBD° (Video S3) starting from the completely dissociated heterodimers. We performed eight SuMD binding replicas for both RBD^Δ^ and RBD°, followed by 200 ns of unsupervised classic MD to allow the complexes produced during SuMD to further relax. The replicas that better reproduced the experimental complex geometry were analysed and compared with RBD^WT^ SuMD simulations from our previous work [23] (Figure 3a–c).

In three SuMD replicas out of the best four, the ACE2:RBD^Δ^ complex reached RMSD values to the final bound complex of 5 Å or less (Figure S3a), in line with equilibrium MD simulations of the ACE2:RBD^Δ^ complex (Figure 3a) while all the best three ACE2:RBD° SuMD binding simulations remained above 5 Å (Figure S3b). To address this difference, we extracted all the MD frames with a binding energy > 5 kcal mol^−1^; the rationale being that the binding kinetics is determined by the energy of the transition states (TSs) along the pathway [54], therefore, the propensity to bind ACE2 can be understood by determining potential transition states along the binding pathway. Importantly, we did not consider the less stable configurations from SuMD simulations as the actual TS of binding, for two reasons. The first is that TSs inherently suffer from poor MD sampling and extensive simulations are required to capture high energy states of the system [55]; the second reason is that our MM-GBSA analysis, which uses an implicit solvent, neglected the explicit entropic contribution to the free energy of binding. It follows that the conformational entropy of the proteins, the roto-translational entropy of water molecules, and the contribution of desolvation to the free energy of binding were not fully taken into account (they are implicit in the SA term). Nevertheless, we assume that frames with an MM-GBSA energy > 5 kcal mol^−1^ can give insight into the enthalpic nature of the TS during the binding to ACE2 and we refer to these frames as unstable states (USs).

As a reference, we used MD data from our previous work [23] regarding RBD^WT^. Binding simulations of the RBD^WT^ occurred through numerous USs (Figure 3a) that were due to destabilising interactions involving D405^WT^, E406^WT^, and Q493^WT^, while K417^WT^, T500^WT^, and T505^WT^ stabilised the binding pathway to ACE2 (Figure 3e). During the binding simulations of all the variants, ACE2:RBD° displayed higher RMSD to the experimental complex than ACE2:RBD^Δ^ (Figure S3a,b), as supported by the broader distribution of US conformations along the binding pathway (Figure 3b–d), suggesting a less efficient propensity to dynamically engage ACE2. RBD° made atomic contacts and hydrogen bonds with ACE2 through Y449° and S446° (Figure S4b,c). RBD^Δ^, instead, made more widespread interactions with the receptor, through N501^Δ^, Y453^Δ^, and K417^Δ^. 

The per-residue binding energy decomposition (Figure 3f,g) highlights the RBD^Δ^ and RBD° residues that stabilised or destabilised the USs during binding. D405^Δ^, E406^Δ^, and D420^Δ^ destabilised the binding of RBD^Δ^, while K417^Δ^ and V503^Δ^ putatively stabilised it, thanks to hydrogen bonds with D30^ACE2^ in the case of K417^Δ^ (Table S4). Moving to RBD°, the most stabilising residues during binding were Y449°, which formed a hydrogen bond with E37 (Table S4) and F486°, while K493° increased the energy of the USs intermediate states (Figure 3g) despite weak hydrogen bonds with E35 and D38 (Table S4). Overall, fewer RBD° residues contributed to binding USs compared to RBD^Δ^. This is in line with the lower RBD°: ACE2 total hydrogen bonds occupancy in USs (Table S4) compared to RBD^Δ^ (118.7 and 152.8, respectively).

Altogether, these results suggest a different binding pathway for RBD^Δ^ and RBD° driven by some of the mutations occurring between the two strains of the virus. Residue K417^Δ^ appears pivotal in orienting the binding to ACE2 by forming the strong hydrogen bond with D30 since the first step of RBD^Δ^ recognition. From this standpoint, the smaller neutral N493^Δ^ side chain is predicted not to affect the binding transition states, compared to K493, which instead destabilised the binding pathway and possibly forced RBD° to engage ACE2 from a different orientation than RBD^Δ^, as shown in Figure 3b,c. Kinetics experiments ruled out any influence of mutation N501Y on the RBD binding on-rate [16]. However, in our simulations, N501^Δ^ formed a stabilising hydrogen bond with K353^ACE2^ in the first steps of RBD^Δ^ binding to ACE2 (Table S4), whilst Y501° was not involved during USs. This inconsistency could have arisen because the kinetic data refer to the single RBD mutant or because of the inherent limits of the MM-GBSA model. 

### 3.3. Omicron Variants

Very recently, other omicron variants, e.g., the SARS-CoV-2 variant BA.2 (also named “Omicron 2” or “stealth Omicron”) emerged as the dominant strain over SARS-CoV-2 Delta and SARS-CoV-2 Omicron [56]. The reasons for its higher infectivity are still under debate. From a structural standpoint, the RBD of BA.2 (RBD^BA.2^) differs from RBD° by six residues: L371F, T376A, D405N, R408S, S446G, and S496G [57]. Only S496 was involved in direct interactions with ACE2 in our equilibrium simulations of the ACE2:RBD°, with K353^ACE2^ and E38^ACE2^ (hydrogen bond occupancy of 18.3% and 13.1%, respectively, Table S4), while S446 formed transitory hydrogen bonds with N330^ACE2^, E37^ACE2^, and R393^ACE2^ in the USs during the binding to the receptor (Table S4). We, therefore, decided to perform SuMD binding simulations of RBD^BA.2^ (Video S4) for comparison with RBD°. During the best three replicas out of eight, RBD^BA.2^ was able to engage ACE2 rapidly and with conformations very close to the experimental complex available for RBD° (Figure S3c) and with a distribution of RBD^BA.2^. conformations from the USs along the binding pathway are more compact than RBD°, as shown in Figure 3d. The reason for this could be found in the smooth interactions with the receptor in the USs (Figure 3h, Table S5, Table S6). Indeed, while F486^BA^. and Y501^BA.2^ are predicted to stabilise RBD^BA.2^ during binding, very weak destabilising contributions are proposed for E406^BA.2^ and K493^BA.2^ in the central part of the domain. The absence of the G496S mutation in BA.2 has been related to the enhanced affinity of BA.2 on the basis that S496 would disturb the local interaction networks of D38^ACE2^ with R/K498 and Y449 [22]. We speculate that the S446G and S496G mutations improve the RBD^BA.2^ binding pathway by allowing higher conformation plasticity than RBD° and therefore favouring the engagement of important residues for binding such as T500^BA.2^, Y501^BA.2^ and H505^BA.2^ (Figure S4c). The conserved RBD residue F486 stabilised the binding pathway of all the variants, suggesting a key role in infectivity. The most recent VOCs BA.4 and BA.5 bear the mutation F486V, which is considered key for antibody escape [58,59]. Therefore, F486V would retain a stabilising contribution during RBD binding in light of the similar hydrophobic properties. This effect of variants on the binding pathway is independent of any effect the variants may have on shifting the up and down conformational equilibrium towards the up form, increasing the probability of ACE2 recognition and, therefore, infectivity. However, this has not yet been reported for Omicron, which shows instead a high number of mutations grouped on the apical part of the RBD, responsible for direct interactions with ACE2 receptors.

## 4. Conclusions

We computationally assessed and compared the binding properties and binding pathway of RBD^Δ^ and RBD° to understand the putative role of key mutations in enhancing the infectivity, despite the binding affinity for ACE2 being almost unchanged, compared with the Delta variant. Our equilibrium simulations of RBD^WT^, RBD^Δ^ and RBD° suggest that RBD°: ACE2 is more stable than the former complexes, thanks to the contribution of new salt bridges formed by K493°; this would lower the RBD° *k_off_*. Our binding pathway simulations show that the same mutation disfavoured the dynamic binding to ACE2 by destabilising the putative USs during the recognition and, therefore, decreased the *k_on_*. The higher stability of the RBD° complex could produce a longer residence time on the receptor, increasing the chances for TMPRSS2 to cleave the S protein and start the membrane fusion, with the final effect of enhancing the infectivity. The even more recent outbreak of the Omicron sub-variant BA.2 could be related to a very fast kinetics of binding to ACE2 thanks to four mutations involving RBD^BA.2^, which optimise the energy profile of the binding pathway resulting in the overall increase in infectivity. We need to bear in mind the inherent limits of MM-GBSA computations [60]; nevertheless, we propose that it is important to understand the dynamic nature of the RBD binding pathway in order to understand infectivity. Future kinetic studies are required to support or confute our findings.

## Data Availability

MD file and trajectories presented in this study are available on request from the corresponding authors.

## Supplementary Materials

The following supporting information can be downloaded at https://www.mdpi.com/article/10.3390/biom12111607/s1. Video S1. PCA analysis of RBDWT:ACE2, RBD:ACE2, and RBDO:ACE2. The first principal component of the Cα coordinates is reported as animation and displays the rocking motion of the RBDs relative to ACE2. Video S2. Representative RBD^Δ^:ACE2 SuMD binding simulation. RBD (blue ribbon and stick representation) recognizes ACE2 (red ribbon and stick) during a SuMD replica. The experimental position and conformation of RBD is reported in transparent grey as reference. Video S3. Representative RBD°: ACE2 SuMD binding simulation. RBDO (blue ribbon and stick representation) recognizes ACE2 (red ribbon and stick) during a SuMD replica. The experimental position and conformation of RBD is reported in transparent grey as reference.Video S4. Representative RBD^BA.2^:ACE2 SuMD binding simulation. RBDBA.2 (blue ribbon and stick representation) recognizes ACE2 (red ribbon and stick) during a SuMD replica. The experimental position and conformation of RBD is reported in transparent grey as reference. Figure S1. The rationale for assigning D206ACE2, E375ACE2, and E489ACE2 as deprotonated. (a) E489 (vdW sphere) side chain is in contact with the positively charged R482. (b) E375 (vdW sphere) is part of Zn-coordinating residues; Zn was removed from the system without changing the protonation states of the coordinating residues. (c) D206 (vdW sphere) is partially solvent exposed (SASA = 46.98 Å3) and in the deprotonated form is destabilized by E398 side chain but stabilized by the K562 side chain. Residues within at least 5 Å are shown as sticks; PDB 6M17. Figure S2. PBSA electrostatic potential. (a) RBD^WT^ and (b) RBD°. The potential is plotted on the molecular surface; the ACE2 binding motif is highlighted within a rectangular shape. Two views are shown for both RBD^WT^ and RBD°. Figure S3. (a) RMSD of RBD^Δ^ to the bound complex over the time course of the best four SuMD replicas; (b) RMSD of RBD° to the bound complex over the time course of the best three SuMD replicas; (c) RMSD of RBD^BA.2^ to the bound complex over the time course of the best three SuMD replicas. Figure S4. RBD^Δ^, RBD° and RBD^BA.2^ contacts and hydrogen bonds with ACE in the unstable states along the binding path to ACE2. (a) Comparison between the intermolecular contacts formed by RBD^Δ^ or RBD° in the unstable states of SuMD binding to ACE2; red residues interacted more in RBD° while blue residues were more engaged in RBD^Δ^. (b) Comparison between the hydrogen bonds formed by RBD^Δ^ or RBD° in the unstable states of SuMD binding to ACE2; red residues interacted more in RBD° while blue residues were more engaged in RBD^Δ^. (c) RBD^BA.2^ contacts in the unstable states of SuMD binding to ACE2; red residues interacted with ACE2, while cyan residues were not engaged. Table S1. Omicron mutations and deletions. Residues within the RBD are in bold. Table S2. Summary of the MD simulations performed. Table S3. MM-GBSA Energy from MD simulations of ACE2 in complex with RBD^WT^, RBD^Δ^, and RBD°. Table S4. Hydrogen Bonds between ACE2 and RBD^Δ^ or RBD° during SuMD binding or classic MD simulations. Residues that differ between RBD^Δ^ and RBD° are in bold. Table S5. Per S1 residue energy contribution to SuMD binding USs. Table S6. Per ACE-2 residue energy contribution to SuMD binding USs.
