# Supplementary material for: A Pathway Model to Understand the Evolution of Spike Protein Binding to ACE2 in SARS-CoV-2 Variants
Source: Biomolecules. 2022 Oct 31;12(11):1607. doi: 10.3390/biom12111607 (PMC9687612; doi:10.3390/biom12111607)
Supplement: Supplementary file 1 [file biomolecules-12-01607-s001.zip › biomolecules-1940881-supplementary-final.pdf]

Supplementary Information

Video S1. PCA analysis of RBD^{WT}: ACE2, RBD^{Δ}: ACE2, and RBD^{O}: ACE2. The first principal component of the $C\alpha$ coordinates is reported as animation and displays the rocking motion of the RBDs relative to ACE2. https://livecoventryac-my.sharepoint.com/:v:/r/personal/ad5291_coventry_ac_uk/Documents/Attachments/Video_S1%203%201.mpg?csf=1&web=1&e=QbB0LI.

Video S2. Representative RBD^{Δ}: ACE2 SuMD binding simulation. RBD^{Δ} (blue ribbon and stick representation) recognizes ACE2 (red ribbon and stick) during a SuMD replica. The experimental position and conformation of RBD is reported in transparent grey as reference. https://livecoventryac-my.sharepoint.com/:v:/r/personal/ad5291_coventry_ac_uk/Documents/Attachments/Video_S2%202%201.mpg?csf=1&web=1&e=Eqc01G.

Video S3. Representative RBD^{O}: ACE2 SuMD binding simulation. RBD^{O} (blue ribbon and stick representation) recognizes ACE2 (red ribbon and stick) during a SuMD replica. The experimental position and conformation of RBD is reported in transparent grey as reference. https://livecoventryac-my.sharepoint.com/:v:/r/personal/ad5291_coventry_ac_uk/Documents/Attachments/Video_S3%201%201.mpg?csf=1&web=1&e=feJXZy.

Video S4. Representative $RBD^{BA.2}$: ACE2 SuMD binding simulation. $RBD^{BA.2}$ (blue ribbon and stick representation) recognizes ACE2 (red ribbon and stick) during a SuMD replica. The experimental position and conformation of RBD is reported in transparent grey as reference. https://livecoventryac-my.sharepoint.com/:v:/r/personal/ad5291_coventry_ac_uk/Documents/Attachments/Video_S4%201%201.mpg?csf=1&web=1&e=ygQDUz.

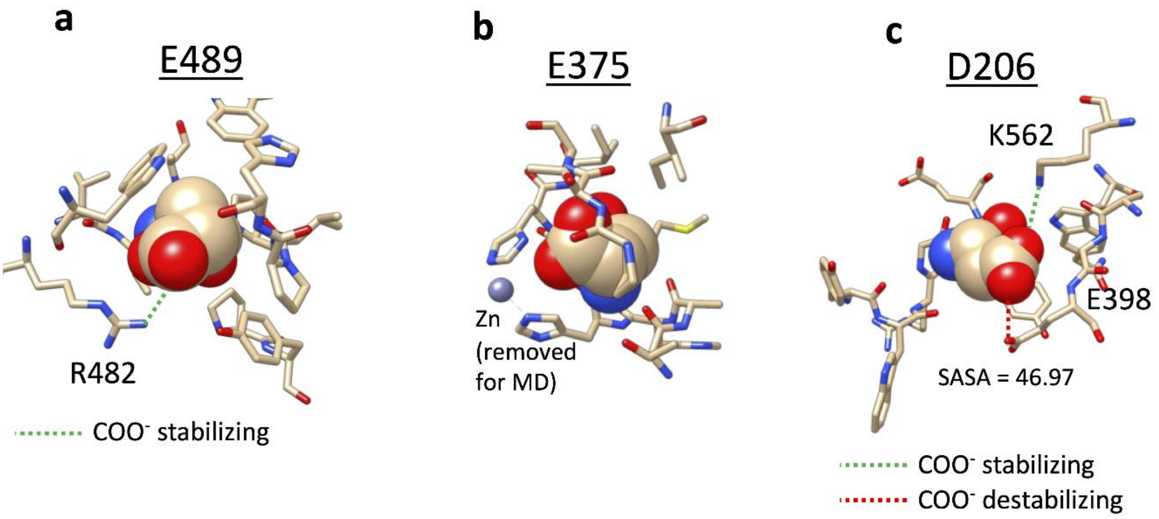

Figure S1. The rationale for assigning D206^{ACE2}, E375^{ACE2}, and E489^{ACE2} as deprotonated. (**a**) E489 (vdW sphere) side chain is in contact with the positively charged R482; (**b**) E375 (vdW sphere) is part of Zn-coordinating residues; Zn was removed from the system without changing the protonation states of the coordinating residues (**c**) D206 (vdW sphere) is partially solvent exposed (SASA = 46.98 Å^3) and in the deprotonated form is destabilized by E398 side chain but stabilized by the K562 side chain. Residues within at least 5 Å are shown as sticks; PDB 6M17.

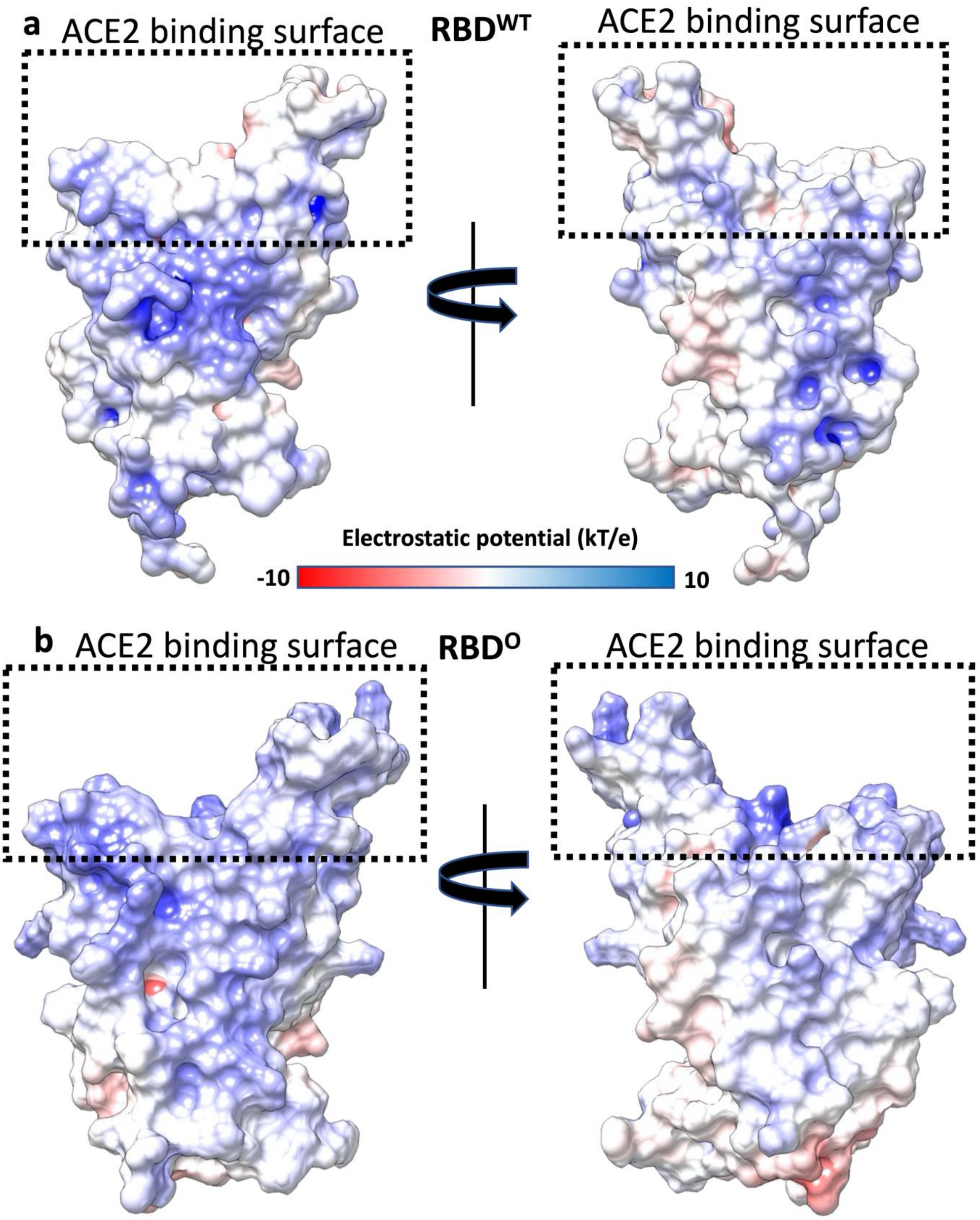

Figure S2. PBSA electrostatic potential. (a) RBD^{WT} and (**b**) RBD^{O}. The potential is plotted on the molecular surface; the ACE2 binding motif is highlighted within a rectangular shape. Two views are shown for both RBD^{WT} and RBD^{O}.

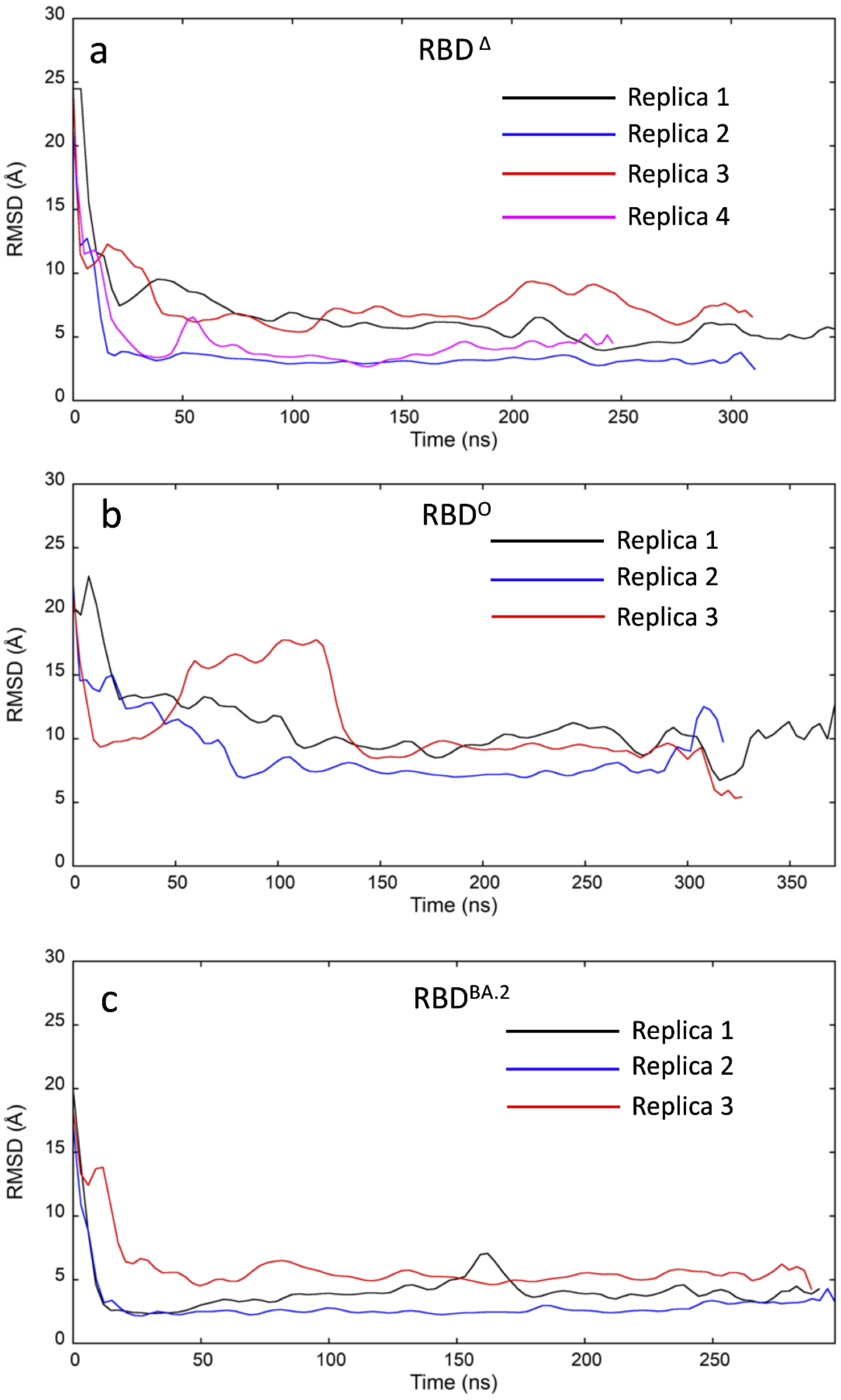

Figure S3. (**a**) RMSD of RBD^{Δ} to the bound complex over the time course of the best four SuMD replicas; (**b**) RMSD of RBD^{O} to the bound complex over the time course of the best three SuMD replicas; (**c**) RMSD of $RBD^{BA.2}$ to the bound complex over the time course of the best three SuMD replicas.

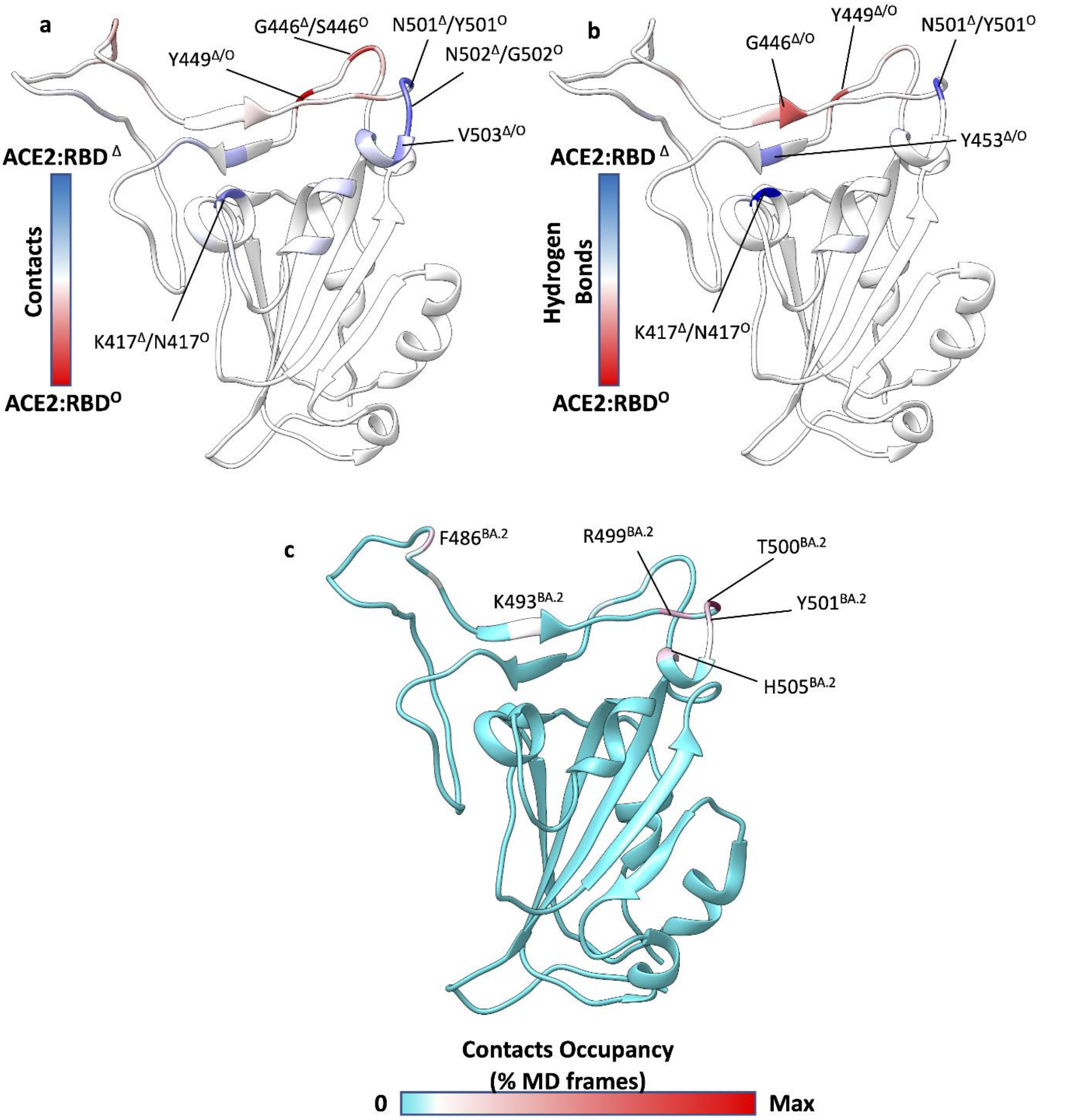

Figure S4. RBD$^{\Delta}$, RBD^{O} and RBD$^{BA.2}$ contacts and hydrogen bonds with ACE in the unstable states along the binding pathway to ACE2. (**a**) Comparison between the intermolecular contacts formed by RBD$^{\Delta}$ or RBD^{O} in the unstable states of SuMD binding to ACE2; red residues interacted more in RBD^{O} while blue residues were more engaged in RBD$^{\Delta}$. (**b**) Comparison between the hydrogen bonds formed by RBD$^{\Delta}$ or RBD^{O} in the unstable states of SuMD binding to ACE2; red residues interacted more in RBD^{O} while blue residues were more engaged in RBD$^{\Delta}$. (**c**) RBD$^{BA.2}$ contacts in the unstable states of SuMD binding to ACE2; red residues interacted with ACE2, while cyan residues were not engaged.

Table S1. Omicron mutations and deletions. Residues within the RBD are in bold.

WT-Omicron mutation	WT-Omicron Deletions
A67V, T95I, Y145D, G339D, ***S371L, S373P, S375F, K417N, N440K, G446S, S477N, T478K, E484A, Q493R, G496S, Q498R N501Y, Y505H,*** *T547K, D614G H655Y, N679K, P681H, N764K D796Y, N856K, Q954H, N969K, L981F*	H69-, V70-, G142-, V143- Y144-, N211-, L3674-, S3675-, and G3676

Table S2. Summary of the MD simulations performed.

System	Simulation Type	# of replicas	Total Simulations length
RBD^{WT}: ACE2	cMD	3	1.5 μs
RBD^{Δ}: ACE2	cMD	3	1.5 μs
RBD^{O}: ACE2	cMD	3	1.5 μs
RBD^{Δ}: ACE2	SuMD + cMD	8	2.53 μs
RBD^{O}: ACE2	SuMD + cMD	8	2.61 μs
$RBD^{BA.2}$: ACE2	SuMD + cMD	8	2.43 μs

Table S3. MM-GBSA Energy from MD simulations of ACE2 in complex with RBD^{WT}, RBD^{Δ}, and RBD^{O}

Complex	Replica	Energy (kcal mol^{-1})	Average (kcal mol^{-1})
**RBD^{WT}:ACE2**	1	-19.8 ± 8.5	
	2	-27.9 ± 5.8	-23.4±4.0
	3	-22.4 ± 6.2	
**RBD^{Δ}:ACE2**	1	-25.3 ± 7.6	
	2	-16.4 ± 7.7	-23.0±4.6

	3	-27.2 $\pm$ 8.4	
**RBD^{O}:ACE2**	1	-29.9 $\pm$ 5.7	
	2	-24.7 $\pm$ 6.5	-28.5$\pm$3.4
	3	-30.9 $\pm$ 6.5	

Table S4. Hydrogen Bonds between ACE2 and RBD$^{\Delta}$ or RBD^{O} during SuMD binding (unstable states, merged replicas) or classic MD simulations (merged replicas). Residues that differ between RBD$^{\Delta}$ and RBD^{O} are in bold.

**Hydrogen Bonds RBD$^{\Delta}$ (USs from SuMD binding)**			**Hydrogen Bonds RBD$^{\Delta}$ (equilibrium complex with ACE2)**			**Hydrogen Bonds RBD^{O} (USs from SuMD binding)**			**Hydrogen Bonds RBD^{O} (equilibrium complex with ACE2)**		
**RBD Res**	**ACE2 Res**	**Occupancy (% MD frames)**	**RBD Res**	**ACE2 Res**	**Occupancy (% MD frames)**	**RBD Res**	**ACE2 Res**	**Occupancy (% MD frames)**	**RBD Res**	**ACE2 Res**	**Occupancy (% MD frames)**
**K417**	D30	43.3	**K417**	D30	79.4	S494	H34	32	N487	Y83	94.3
**N501**	K353	38.6	T500	D355	62.4	Y449	E37	32	**K493**	E35	91.5
**Y453**	H34	21.9	**Q493**	E35	48.8	**K493**	E35	10.1	T500	D355	85.9
**K417**	T27	8.1	**Y505**	E37	37.1	**K493**	D30	6.5	**K493**	D38	67.3
**Y473**	Q24	6.1	Y453	H34	35.7	N450	D38	5.3	T500	Y41	27.5
**R408**	Q388	4.8	T500	Y41	29.3	**S446**	N330	4.7	N487	Q24	27
**Y505**	H34	4.8	**Q498**	K353	27.4	Y489	Q24	4.7	Y453	H34	27
**Q493**	H34	4.4	**Q498**	Q42	22.6	**S446**	E37	3.6	**R498**	Q42	26.6
**Y421**	E23	3.6	**Q493**	K31	22.5	**R498**	D30	3	**S496**	K353	18.3
**T415**	E23	2.8	Y449	D38	21.9	**R498**	E329	3	**S496**	D38	13.2
**Y489**	Q24	2.7	N487	Q24	20.2	**K493**	D38	3	**Y501**	K353	10
**Q493**	K31	2.3	N487	Y83	17.7	Y489	S19	2.4	**N477**	Q24	8
**Y489**	Y83	1.9	**N501**	Y41	17.4	**R498**	E37	1.8	Y449	D38	6.7
**R403**	H34	1.7	Y489	Y83	9	**S446**	R393	1.8	**N477**	S19	6.4
**Q409**	K26	1.7	**Q493**	H34	5.5	N487	E35	1.2	**N477**	T20	5.3

**Q498**	N64	1.7	**Y505**	R393	5	**K493**	H34	1.2
**Q493**	E35	1.3				Y449	H34	1.2
**Y505**	E37	1.1				Y489	E75	1.2

Table S5. Per S1 residue energy contribution to SuMD binding USs.

RBD Residue	WT / kcal mol^{-1}	Delta / Kcal mol^{-1}	Omicron / kcal mol^{-1}	BA.2 / kcal mol^{-1}
**$D405^{WT}/D405^{\Delta}/D405^{O}/N405^{BA2}$**	0.75 ± 0.57	2.13 ± 0.90	0.10 ± 0.10	0.14 ± 0.12
**$D420^{WT}/D420^{\Delta}/D420^{O}/D420^{BA2}$**	0.15 ± 0.16	1.37 ± 1.20	0.03 ± 0.04	0.06 ± 0.08
**$E406^{WT}/E406^{\Delta}/E406^{O}/E406^{BA2}$**	0.36 ± 0.32	1.29 ± 0.66	0.23 ± 0.13	0.42 ± 0.23
**$E484^{WT}/E484^{\Delta}/A484^{O}/A484^{BA2}$**	0.25 ± 0.26	0.21 ± 0.27	-0.09 ± 0.34	0.00 ± 0.08
**$F456^{WT}/F456^{\Delta}/F456^{O}/F456^{BA2}$**	-0.19 ± 0.36	-0.40 ± 0.59	-0.04 ± 0.21	-0.12 ± 0.43
**$F486^{WT}/F486^{\Delta}/F486^{O}/F486^{BA2}$**	-0.14 ± 0.36	-0.65 ± 1.18	-1.68 ± 1.57	-1.17 ± 1.28
**$G446^{WT}/G446^{\Delta}/S446^{O}/G446^{BA2}$**	-0.09 ± 0.31	0.04 ± 0.03	0.56 ± 0.88	0.07 ± 0.08
**$G485^{WT}/G485^{\Delta}/G485^{O}/G485^{BA2}$**	0.09 ± 0.10	0.08 ± 0.09	-0.35 ± 0.66	0.05 ± 0.24
**$G502^{WT}/G502^{\Delta}/G502^{O}/G502^{BA2}$**	-0.94 ± 0.76	-0.33 ± 0.40	0.04 ± 0.02	0.08 ± 0.19
**$G504^{WT}/G504^{\Delta}/G504^{O}/G504^{BA2}$**	0.14 ± 0.16	-0.34 ± 0.39	0.01 ± 0.01	0.01 ± 0.03
**$K417^{WT}/K417^{\Delta}/N417^{O}/N417^{BA2}$**	0.91 ± 1.47	-0.67 ± 2.70	0.03 ± 0.03	0.06 ± 0.06
**$K444^{WT}/K444^{\Delta}/K444^{O}/K444^{BA2}$**	0.15 ± 0.15	0.02 ± 0.02	0.65 ± 0.67	0.11 ± 0.05
**$L455^{WT}/L455^{\Delta}/L455^{O}/L455^{BA2}$**	-0.04 ± 0.18	-0.55 ± 0.75	-0.16 ± 0.33	-0.12 ± 0.31
**$N501^{WT}/N501^{\Delta}/Y501^{O}/Y501^{BA2}$**	-0.02 ± 0.87	-0.96 ± 1.38	0.35 ± 0.25	-0.76 ± 1.36
**$Q493^{WT}/Q493^{\Delta}/K493^{O}/K493^{BA2}$**	0.18 ± 0.47	-0.24 ± 0.64	1.21 ± 2.17	0.40 ± 0.99
**$Q498^{WT}/Q498^{\Delta}/R498^{O}/R498^{BA2}$**	0.39 ± 0.93	0.1 ± 0.32	0.15 ± 1.28	0.42 ± 1.03
**$Q506^{WT}/Q506^{\Delta}/Q506^{O}/Q506^{BA2}$**	0.57 ± 0.23	0.14 ± 0.11	0.00 ± 0.01	0.02 ± 0.04
**$R403^{WT}/R403^{\Delta}/R403^{O}/R403^{BA2}$**	0.04 ± 1.07	-0.47 ± 0.71	0.04 ± 0.18	0.01 ± 0.11
**$R408^{WT}/R408^{\Delta}/R408^{O}/S408^{BA2}$**	0.21 ± 0.25	0.26 ± 0.44	-0.01 ± 0.03	0.04 ± 0.04
**$S443^{WT}/S443^{\Delta}/S443^{O}/S443^{BA2}$**	0.23 ± 0.17	0.08 ± 0.03	0.06 ± 0.05	0.05 ± 0.02
**$T500^{WT}/T500^{\Delta}/T500^{O}/T500^{BA2}$**	-0.96 ± 1.09	-0.59 ± 1.12	0.07 ± 0.17	-0.22 ± 1.07
**$V445^{WT}/V445^{\Delta}/V445^{O}/V445^{BA2}$**	-0.42 ± 0.73	0.03 ± 0.05	-0.46 ± 0.74	-0.05 ± 0.23
**$V483^{WT}/V483^{\Delta}/V483^{O}/V483^{BA2}$**	0.02 ± 0.03	0.01 ± 0.02	-0.47 ± 0.87	0.01 ± 0.01

**$V503^{WT}/V503^{\Delta}/V503^{O}/V503^{BA2}$**	0.14 ± 0.11	-1.58 ± 1.23	0.00 ± 0.00	-0.21 ± 0.29
**$Y449^{WT}/Y449^{\Delta}/Y449^{O}/Y449^{BA2}$**	-0.06 ± 0.42	0.14 ± 0.10	-1.94 ± 1.99	0.27 ± 0.23
**$Y453^{WT}/Y453^{\Delta}/Y453^{O}/Y453^{BA2}$**	0.05 ± 0.18	0.36 ± 0.42	0.18 ± 0.11	0.18 ± 0.12
**$Y505^{WT}/Y505^{\Delta}/H505^{O}/H505^{BA2}$**	-1.77 ± 1.67	-0.21 ± 0.51	0.18 ± 0.13	0.19 ± 0.94

Table S6. Per ACE2 residue energy contribution to SuMD binding USs.

**ACE-2 Residues**	**WT / kcal mol^{-1}**	**Delta / kcal mol^{-1}**	**Omicron / kcal mol^{-1}**	**BA.2 / kcal mol^{-1}**
**Y41**	-0.38 ± 0.64	-0.16 ± 0.22	0.03 ± 0.17	-0.77 ± 0.72
**T27**	0.04 ± 0.15	-0.86 ± 1.46	-0.13 ± 0.48	-0.12 ± 0.42
**S19**	N/A	0.88 ± 1.07	1.11 ± 0.80	0.75 ± 0.61
**Q42**	-0.61 ± 1.15	0.04 ± 0.21	0.08 ± 0.06	-0.43 ± 0.64
**M82**	-0.09 ± 0.27	-0.32 ± 0.65	-0.88 ± 1.01	-0.31 ± 0.59
**L79**	-0.07 ± 0.21	-0.38 ± 0.66	-0.62 ± 0.75	-0.18 ± 0.4’
**L45**	-0.58 ± 0.65	-0.04 ± 0.08	0.00 ± 0.01	-0.52 ± 0.32
**K68**	1.55 ± 1.48	0.16 ± 0.34	0.31 ± 0.38	0.41 ± 0.29
**K353**	0.42 ± 1.25	-0.39 ± 1.26	1.04 ± 1.08	0.65 ± 0.90
**K31**	0.99 ± 1.06	0.32 ± 0.95	1.41 ± 1.60	1.17 ± 1.10
**K26**	0.19 ± 0.27	1.71 ± 1.37	0.64 ± 0.95	0.19 ± 0.15
**H34**	-0.12 ± 0.86	0.1 ± 0.92	-0.41 ± 1.03	0.41 ± 0.32
**G354**	-0.23 ± 0.43	-0.72 ± 0.50	-0.18 ± 0.33	-0.04 ± 0.16
**E75**	0.5 ± 0.46	0.25 ± 0.31	0.73 ± 0.84	0.2 ± 0.27
**E37**	-0.01 ± 0.59	1.45 ± 0.77	1.12 ± 1.29	0.25 ± 0.19
**E35**	0.86 ± 0.50	0.93 ± 0.55	1.25 ± 1.06	0.47 ± 0.69
**E329**	0.2 ± 0.32	0.15 ± 0.12	0.02 ± 0.40	0.41 ± 0.33
**E23**	0.11 ± 0.21	1.96 ± 1.67	0.33 ± 0.37	0.21 ± 0.19
**D38**	0.41 ± 1.10	1.28 ± 0.74	1.5 ± 1.15	1.24 ± 0.81
**D355**	-0.36 ± 0.66	0.39 ± 0.24	0.13 ± 0.18	0.48 ± 1.17
**A387**	-0.02 ± 0.16	-0.82 ± 0.97	-0.46 ±0.72	0.02 ± 0.01
